# Child Life under Queen Victoria

**Published:** 1897-04-10

**Authors:** 


### Child Life under Queen Victoria.

### IX.—CANAL-BOAT CHILDREN.

The advent of railways has almost made us forget those winding waterways by which in the early years of the century so much heavy merchandise was conveyed. But even now the canals are more used for the carriage of heavy stuff than many of us realise. To the outside world the canal boat, with the patient, laborious tow-horse, the slow moving boat making sluggish ripples on the still water, the master lazily steering along the familiar route—the canal boat as it moves among the fields is one of the most picturesque objects in a land-scape which the noisy railway tends to make common-place. At a distance the canal boat is attractive, but on a nearer view, whether empty or laden with clay, bricks, vile-smelling manure, or any other of its cargoes, the canal boat is, considered as a family home, unattractive and unsatisfactory.

On board that heavy barge, sleeping at night in that cramped cabin, a whole family, father, mother, and children, is living—how? In America, where the canals form a vaster network and the journeys are longer than here, these boats are too often the home of nameless vices. But even here, in that languid wandering from one town to another, with no settled abiding-place, what chance was there of bringing up children that would be useful citizens? From generation to generation they would go on, sluggish and illiterate, knowing no world but the wharf and the tow-path.

The canal boat of the past was an overcrowded, unhealthy hut, where parents and children of all ages huddled promiscuously together, and grew up in ignorance and heathenism. Voluntary missionary effort did what it could to inculcate the principles of religion and decency; but the best voluntary effort can only affect those who are by nature more or less prepared for improvement. It takes the law of the land to enforce so much respect for at least ordinary proprieties as shall, at the end of a generation, make the new race wonder at the grossness and brutality of their fathers. Law cannot do everything; in the most spiritual sense cannot do much for any human soul, but it can remove many spiritual hindrances which interfere with the soul's growth. Thus, no law can make the master of a canal boat clean in habits, pure in life, considerate to his children, but by restrictions here and punishment there it can make it more profitable to him to cultivate these moralities than to ignore them.

It was to this end that in 1877 the Canal Boats Act was passed. It was therein enacted that all canal boats which are used as dwellings should be registered, and the number, ages, and sexes allowed to occupy it regulated according to the cubic space, ventilation, general healthiness, and convenience of accommodation on the boat. Once investigated and registered the boat was free; but if there was suspicion that there was a case of infectious disease on board any canal boat, the sanitary authority of the neighbourhood was authorised to go on board and investigate. If an infectious case was found the patient could be removed, and, moreover, the boat was to be detained until it was thoroughly cleansed and disinfected.

In connection with the education of canal-boat children a difficulty was bound to arise. With people of such a class as the canal boatmen it is not enough to insist on education without fixing the place where the child is to be educated. To this end it was fixed that children should be registered as belonging to the place at which their parents' boat was registered. There they were to attend school, unless it could be shown that due provision had been made for their education elsewhere. A special paragraph in the Act authorised canal companies, or any association of owners of canal boats, to establish schools for the maintenance of schools where the children of canal boatmen might be boarded, lodged, and educated. The lodging and education are allowed to be gratuitous, but in order to keep up the due sense of parental responsibility the parents have to pay for their children's maintenance.

It is obvious that with itinerant trades such as this it is difficult to get such a hold upon parents as to compel them to do justice to their children; but the regulations as to overcrowding have done much to minimise the evils of such a life. Parents do leave their children in the port of registry with relations or friends. There they attend school; they are surrounded by varied influences, not all good it may be, but at any rate fit to make them realise that there is a bigger world than the lazy boat on the sluggish canal. Their parents may indeed take them too often for a trip on the boat—no unwelcome holiday for the children—and break the course of schooling; but they no longer dwell in that isolation which made the canal boatman as isolated as any Indian caste.

For other classes something similar remains to be done—for tinkers and travelling show folk—for all itinerants in fact. Among them one still meets with children who have never been to school nor church, have no ideal of life beyond the sordid, often dishonest round their parents know. But all these people are more or less stationary in the winter, and then the powers which the law now possesses with regard to dwellings and education may be enforced, and new ideas introduced into the plastic minds of children. There are also at most canal ports cheerful mission-rooms where yet higher ideas are taught. Allowing for the confessed difficulty of dealing with and keeping under surveillance our itinerant population, it may be said that canal boatmen are now compelled to be decent and pressed to be good.

